# Leapfrog diagnostics: Demonstration of a broad spectrum pathogen identification platform in a resource-limited setting

**DOI:** 10.1186/1478-4505-10-22

**Published:** 2012-07-04

**Authors:** Tomasz A Leski, Rashid Ansumana, Anthony P Malanoski, David H Jimmy, Umaru Bangura, Brian R Barrows, Morie Alpha, Bashiru M Koroma, Nina C Long, Abu J Sundufu, Alfred S Bockarie, Baochuan Lin, David A Stenger

**Affiliations:** 1 Center for Bio/Molecular Science and Engineering, Naval Research Laboratory, Washington DC, 20375, USA; 2 Mercy Hospital Research Laboratory, Kulanda Town, Bo, Sierra Leone; 3 Liverpool School of Tropical Medicine, University of Liverpool, Liverpool, UK; 4 Institute of Environmental Management and Quality Control, Njala University, Njala, Sierra Leone; 5 Chemistry Department, Njala University, Njala, Sierra Leone; 6 National Research Council (NRC) Associate, Naval Research Laboratory, Washington DC, 20375, USA; 7 Nova Research Incorporated, Alexandria, VA, 22308, USA; 8 Biology Department of School of Environmental Sciences, Njala University, Njala, Sierra Leone

## Abstract

**Background:**

Resource-limited tropical countries are home to numerous infectious pathogens of both human and zoonotic origin. A capability for early detection to allow rapid outbreak containment and prevent spread to non-endemic regions is severely impaired by inadequate diagnostic laboratory capacity, the absence of a “cold chain” and the lack of highly trained personnel. Building up detection capacity in these countries by direct replication of the systems existing in developed countries is not a feasible approach and instead requires “leapfrogging” to the deployment of the newest diagnostic systems that do not have the infrastructure requirements of systems used in developed countries.

**Methods:**

A laboratory for molecular diagnostics of infectious agents was established in Bo, Sierra Leone with a hybrid solar/diesel/battery system to ensure stable power supply and a satellite modem to enable efficient communication. An array of room temperature stabilization and refrigeration technologies for reliable transport and storage of reagents and biological samples were also tested to ensure sustainable laboratory supplies for diagnostic assays.

**Results:**

The laboratory demonstrated its operational proficiency by conducting an investigation of a suspected avian influenza outbreak at a commercial poultry farm at Bo using broad range resequencing microarrays and real time RT-PCR. The results of the investigation excluded influenza viruses as a possible cause of the outbreak and indicated a link between the outbreak and the presence of *Klebsiella pneumoniae*.

**Conclusions:**

This study demonstrated that by application of a carefully selected set of technologies and sufficient personnel training, it is feasible to deploy and effectively use a broad-range infectious pathogen detection technology in a severely resource-limited setting.

## Background

Developing countries in tropical regions of the world are the home for numerous important infectious pathogens [1-3]. Many of these infectious agents may have their reservoirs in domesticated or wild animals [4-8]. Since inhabitants of these countries live in relatively closer contact with animals, than populations of highly developed countries, the chance of transmission of zoonotic infections to humans is much greater [9]. Some of these pathogens not only have severe impact on public health in countries where they are endemic, but may also be rapidly disseminated to non-endemic regions through global transportation networks (air, freight containers), migratory birds, and expanding wildlife trade [10-15]. While outbreaks caused by highly lethal pathogens such as Ebola, Nipah or H5N1 influenza viruses are usually followed by high profile epidemiologic investigations, the everyday infectious disease diagnostics and epidemiological surveillance systems in many of these regions are only rudimentary, sub-Saharan Africa being one of the prime examples [16,17]. This situation is compounded by serious shortages of resources and trained personnel capable of performing diagnostic procedures. As a consequence, infectious disease outbreaks in these settings are detected relatively late in their course. A typical example of the problem was the recent yellow fever outbreak that began in October 2010 in Uganda. The outbreak, initially suspected to be caused by Ebola virus, started in early October and was subsequently misdiagnosed as amoebic dysentery, alcohol poisoning, and plague before being correctly identified as yellow fever at the end of December. By the time of confirmation, there were approximately 200 confirmed cases and nearly 50 deaths were recorded [18,19].

Rapid and efficient infectious disease surveillance systems are necessary to improve outbreak management and mitigate the consequences of outbreaks. However, direct replication the infectious outbreak surveillance systems in their current form that exist in developed countries is not practical due to many reasons including absence of detailed maps and lack of basic laboratory infrastructure needed to support traditional diagnostic systems. Our previous work on participatory mapping and surveying methods has indicated promising solutions to the first problem [20]. The issues related to inefficiency of current infectious disease diagnostics based on culture and simple molecular assays remain a serious challenge. Microbial culture, while still a “gold standard” technique for identification of bacteria, cannot be applied for detection of viral pathogens. In addition, microbial culture is labor intensive, time consuming and requires qualified and experienced technicians which are frequently in short supply in these locations. Molecular assays are rapid and sensitive but due to their low level of multiplexing usually a number of different molecular assays needs to be performed sequentially to achieve definitive diagnosis. This is especially relevant in cases of infectious syndromes of diverse etiologies but manifested by similar symptoms [21]. Reliance on these technologies results in significant delays between specimen isolation and pathogen identification. An additional difficulty experienced by many regions of the developing world is the lack of a reliable cold chain capability necessary for transport and preservation of biological samples and diagnostic reagents in hot climate due to unreliable power and a lack of basic refrigeration equipment.

One of the ways to significantly improve the microbial diagnostic capacity in developing countries may be ‘leapfrogging’ or skipping some stages of technological development that other countries have passed or are passing through [22,23]. A good example of the “leapfrog” phenomenon is the use of mobile phone technology, which has enabled the communication in villages in developing countries that have never had land phones with their associated expensive infrastructure [24]. In case of microbial diagnostics, developing countries may need to jump directly to broad-range microbial diagnostic systems, which are capable of one-step detection and identification of large number of diverse pathogens in a single, highly automated, assay. Advanced broad-range diagnostic technologies have the potential of making the pathogen identification process simpler and faster leading to more efficient detection and management of infectious disease outbreaks both in humans and animals.

The purpose of this study was to test the feasibility of application of a broad-spectrum diagnostics/surveillance platform for microbial detection in a resource-limited setting. This feasibility study was the result of a collaborative effort between the US Naval Research Laboratory and Njala University, which led to the establishment of a molecular diagnostic laboratory at Mercy Hospital Research Laboratory, Bo, Sierra Leone.

Setting up this type of facility required solving a number of issues typical for developing countries with a tropical climate including securing a reliable power supply, implementing cold chain and complementary methods of preserving biological samples and reagents, and enabling efficient communication by setting up an internet linked computer network. This paper describes how a broad spectrum diagnostics system was successfully deployed using a set of “leapfrog” technologies that were found to be critical in establishing an efficient laboratory. The resequencing-microarray-based diagnostic system was subsequently applied for investigation of a suspected avian influenza outbreak at the commercial poultry farm.

## Methods

### Laboratory setup

The molecular diagnostic laboratory was set up in Mercy Hospital Research Laboratory (MHRL) located in the city of Bo, Sierra Leone. Bo is the second largest city in Sierra Leone and the capital of the Southern Province. The 1,200 square foot laboratory was located on the Mercy Hospital campus in Kulanda Town section of Bo. All equipment, which required temperature within certain limits for proper operation (including PCR instruments, Affymetrix fluidics stations, hybridization ovens and microarray scanner) was located in two air-conditioned rooms. An additional air-conditioned laboratory area detached from the main laboratory building was equipped with a PCR laminar flow hood and used for sample preparation for PCR, RT-PCR and sample processing for resequencing pathogen microarray (RPM) assays.

For cold storage, the laboratory was equipped with two 57 liter AcuTemp AX56L/HemaCool mobile refrigerator/freezers (AcuTemp, Dayton, OH), four Fridge-Freeze 60 liter portable vaccine refrigeration units (two freezers and two refrigerators) with ability to be powered with 12/24-volt DC or 110/240-volt AC (Fridge-Freeze Inc. San Diego, CA) and one upright Kenmore freezer model 2804 (Sears, Roebuck and Co., Hoffman Estates, IL). HemaCool freezers can be adjusted for freezing (−20°C) or refrigeration (+4°C) and can be run for 16 hrs continuously in the absence of external power on internal batteries.

The power for the laboratory operation was supplied by two hybrid power subsystems, one operating at 230 V, 50 Hz (used for powering European and African made equipment) and another one operating at 120 V, 60 Hz (for powering US made scientific instruments). Both systems relied on combination of solar power with battery storage and diesel generator backup. The 230 V subsystem was additionally connected to municipal power grid and used municipal power when available. The detailed description of the power system and its performance was published previously [25,26].

### Communication

A stand-alone solar powered C-band satellite communication system was configured by Satcom Resources (Avon, CO) and deployed at MHRL. Bandwidth (512/128 (kb/s) up/down) was provided by Constellation Networks Corp. (Traverse City, MI) and served up to 20 users simultaneously via intranet across the Mercy Hospital campus. The diagram of the network configuration is included in supplementary data (Additional file 1: Figure S4). Teleconferencing for training purposes and data exchange was conducted using Skype platform (Skype Technologies S.A., Rives de Clausen, Luxembourg).

### Ambient temperature stable reagents

Ambient temperature stabilized reagents for PCR, real-time PCR or resequencing microarray protocols were either obtained commercially or developed in-house using lyophilization techniques (see Additional file 2: Tables S3 and S4 for listing of stabilized reagents tested and used for all molecular diagnostics protocols).

### FTA paper - sample stabilization and recovery

Flinders Technology Associates filter paper (FTA paper, Whatman/GE Healthcare, Florham Park, NJ) [27] was used in this work to explore its suitability for dry storage of RNA preparations and for stabilization of field samples collected from poultry. Either Indicating FTA Mini Cards (WB120356, Whatman) or Indicating FTA Classic Cards (WB120206, Whatman) were used depending on the application. An aliquot of RNA samples was spotted on the card or swabs containing field samples were pressed against an FTA card in order to transfer the maximum amount of fluid from swab to the FTA paper. Subsequently the FTA cards were air dried at ambient temperatures for 40 minutes. Dry FTA cards were stored at ambient temperatures unless otherwise indicated.

To recover the nucleic acids for use in diagnostic procedures, the FTA paper embedded samples were processed using the following procedure modified from a protocol developed by Rogers and Burgoyne [28]. Circular punches (1 mm or 3 mm in diameter) of the FTA paper from the areas of sample deposition were taken using Harris Uni-Core punch (Ted Pella Inc., Redding, CA). The punches were placed into 0.5 mL microcentrifuge tubes and incubated for 5 min. with gentle shaking in 200 μl of solution A (4 M LiCl solution in 50% ethanol). The incubation was repeated once with fresh solution A. After incubation, the disks were washed with gentle shaking for 5 min. in 200 μl of solution B (50 mM Tris–HCl solution in isopropanol), then washed twice for 5 min. with 200 μl of 70% ethanol. After the final wash, the samples were air dried at 42°C for at least 30 minutes to remove the traces of ethanol completely.

### RNA purification

The RNA was purified from influenza B/Lee/40 preparations (Advanced Biotechnologies, Inc., Columbia, MD) using MasterPure DNA and RNA purification kit (Epicentre Biotechnologies, Madison, WI) using manufacturer recommended protocol.

### Testing viral RNA stabilization efficiency on FTA paper

Influenza B RNA samples (5 μl each of 10^6^, 10^4^ or 10^2^ genome copies/μl dilutions) were spotted on Indicating FTA Mini Cards (Whatman). The cards were incubated under one of three conditions: at room temperature 20-25°C (on a laboratory bench), 30°C (incubator) and 30-45°C (outdoors, protected from light) for up to 10 days. Three 1 mm punches were removed from the FTA cards at 24 hours, 3 days, and 10 days). The punches were processed as described above (see “FTA paper - sample stabilization and recovery” section).

### Collection of poultry pharyngeal samples

**Figure 1:**
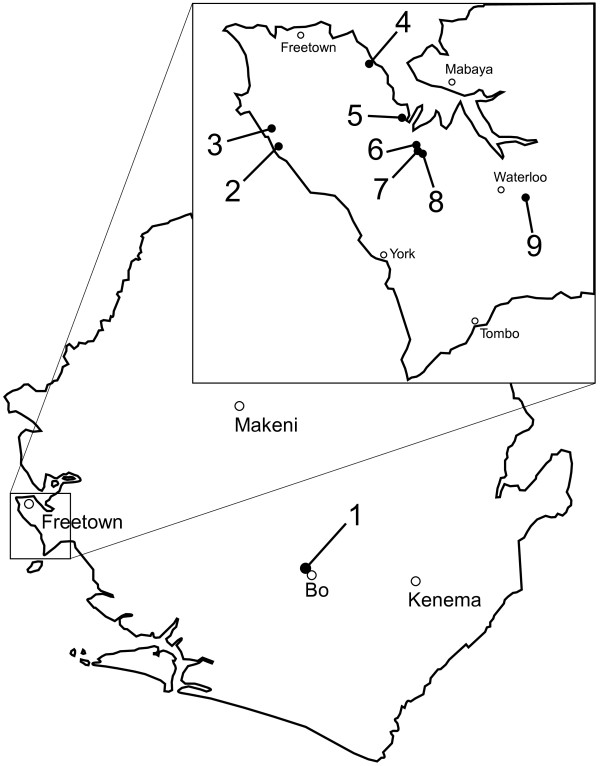
Locations of farms in Sierra Leone where poultry samples were collected.

Pharyngeal swabs were collected on March 30^th^, 2009, using sterile techniques from 136 chickens housed in 9 different poultry farms. The farms were located in Bo and in the vicinity of Freetown (Figure 1), their geographic coordinates were determined using handheld Garmin GPSMAP 60CSx GPS unit (Garmin International Inc., Olathe, KS). Basic farm information, the numbers of samples taken at each farm and general health characteristics of the poultry on farms sampled are included in Table 1. The collected swab samples were stabilized and stored on FTA cards. After transporting to the laboratory in ambient temperatures, the FTA embedded specimens were placed in −20°C freezer for long term storage.

**Table 1:** Farm and chicken sample information

**No.**	**Farm location**		**No. of sheds**	**No. of samples collected**	**Sample designations**	**Condition of chickens**
	**Town**	**Coordinates**				
1	Bo	7°57.911'N 11°44.767'W	1	40	B1-B40	Outbreak affecting 15% of chickens
2	Hamilton	8°23.275'N 13°15.453'W	4	20	H1-H20	All healthy
3	Oogu	8°24.256'N 13°15.612'W	1	16	O1-O16	All healthy
4	Wellington	8°27.222'N 13°10.242'W	3	12	W1-12	Some chickens sick
5	Allen Town	8°24.266'N 13°8.742'W	11	16	A1-16	All healthy
6	Hastings	8°22.776'N 13°8.094'W	1	8	HA1-HA8	All healthy
7	Hastings	8°22.622'N 13°7.997'W	1	4	HB1-HB4	All healthy
8	Hastings	8°22.403'N 13°7.925'W	2	8	HC1-HC8	All healthy
9	Joe Town	8°19.549'N 13°2.535'W	3	12	J1-12	Some chickens sick

### Resequencing microarrays

The resequencing microarray analysis was conducted using RPM-Flu v. 3.1 (RPM-Flu) and RPM-TEI v. 1.0 microarrays (RPM-TEI) in March 2010. Poultry samples for analysis were selected using a two-stage process. First the samples for which the amount of deposited samples was small (judged by the size of spot with change of color on indicating FTA card) were rejected. The remaining FTA samples were blindly drawn to select 15 samples from Bo farm and 3 samples each from 5 other farms. RPM-Flu v. 3.1 was designed to detect all known subtypes of influenza A viruses and 84 other viral and bacterial respiratory pathogens [29], while the RPM-TEI microarray was designed for detection of a broad range of biothreat agents [30] some of them (such as Lassa virus) endemic to West Africa. Sample processing was conducted as previously described [30-32] with the following modifications related to the use of FTA paper embedded samples. Briefly, reverse transcription using random primers was used to obtain cDNA from RNA templates potentially present in a processed FTA paper disk for each analyzed sample. The resulting mixture was separated from the FTA paper disk and split into four aliquots of equal volume for multiplex PCR reactions using either RPM-Flu or RPM-TEI microarray specific primer cocktails. Modified version of this protocol for testing the lyophilized/ambient temperature stabilized reagents is included as Supplementary Data.

Pathogen identification was performed using previously developed Computer-Implemented Biological Sequence Identifier (CIBSI) 2.0 software [33]. Although the microarrays are designed with tiles for specific pathogen targets [29,30,32], they are capable of detection and correct sequence determination of targets differing by up to 15% from the sequence present on the microarray. This allows for detection of target variants and near-neighbor discrimination.

### Reverse transcription and real time PCR (RT-PCR)

In order to detect influenza B RNA in testing RNA stabilization on FTA 1 mm processed disks were placed in a 0.5 mL PCR tube, and subjected to one step reverse transcription and PCR (RT-PCR) using Qiagen OneStep RT-PCR Kit (Qiagen, Valencia, CA) according to the manufacturer’s instructions. Positive controls were prepared using liquid preparations of influenza B RNA stored at −20°C. The RT-PCR reaction was run in 25 μl total volume using previously published BMA-F1 and BMA-R1 primers for detection of influenza B [31] and the following thermal cycling protocol: 50°C for 30 min; 95°C for 15 min.; 40 cycles of 94°C for 30 sec., 54°C 30 sec., 72°C 30 sec.; 72°C 10 min. The expected amplicon was a 162 bp segment of the influenza B matrix gene. The amplification results were analyzed using 2% TAE agarose gels containing ethidium bromide. The bands were visualized in UV light and images captured using UVP BioDoc-It System, model M-20 (UVP, Upland, CA).

Real-time RT-PCR for universal detection of influenza A (based on detection of fragment of the Matrix gene) was performed using previously published PCR primers: MatrixF1 and MatrixR1 [34]. A preparation of influenza A H3N2 control strain was used as a positive control, water and blank processed FTA paper discs were used as negative controls. Reverse transcription (RT) was performed using AccuPower Cyclescript RT premix (Bioneer, Alameda, CA). The total volume of the RT reaction was 20 μl containing 500 nM each of forward and reverse primers. The reaction mixture was subjected to 12 cycles of incubation at 25°C for 1 min. and 50°C for 4 min. and single final incubation at 95°C for 5 min. The real-time PCR amplification reactions were conducted using SsoFast EvaGreen reaction mix (Bio-Rad Laboratories, Hercules, CA) according to the manufacturer’s instructions. The reaction was carried out in 20 μl total volume containing 500 nM of each primer with 2 μl of the RT reaction mixture as a template. The thermal cycling and fluorescent signal detection was performed in a CFX96 real-time PCR detection system (Bio-Rad Laboratories) with the following thermal cycling conditions: initial incubation at 98°C for 2 min. followed by 40 cycles of 98°C for 2 sec. and 60°C for 5 sec. The amplification cycle was followed by melting curve analysis. The results were analyzed with CFX Manager software ver. 1.5.534.0511 (Bio-Rad Laboratories).

The protocols for RT-PCR and real-time RT-PCR for detection of influenza A using ambient temperature stabilized reagents are described in Supplementary Data (Additional file 2), and the reagents used with these protocols are listed in (Additional file 2): Tables S3 and S4.

## Results and discussion

### Laboratory operation

This study was intended to identify the suite of technologies necessary to deploy and successfully apply an advanced, “leapfrogging”, technology for broad range pathogen identification in a severely resource-limited setting. The typical major challenges in setting up a laboratory in a developing country are listed in Table 2. They needed to be addressed in order to make the laboratory operational and capable of reliably running high quality molecular diagnostic protocols. While the general class of solutions was easy to recognize, the actual solution implemented depended intimately on the local conditions.

**Table 2:** Technologies applied to deploy broad-range infectious pathogen diagnostics

**Problem**	**Solution/s applied**
Unreliable power	Efficient hybrid solar/diesel power system
Lack of “cold chain”	On-site refrigeration
	FTA paper for sample collection, transport and storage
	Ambient temperature stabilized reagent sets
	Delivery of regular reagents on dry-ice
Lack of efficient communication	Wired/wireless, hi-speed campus network connected with Internet via satellite
Inadequate personnel expertise	Hands-on training in reference laboratory (NRL) and on-site.
	Remote technical support by email and Skype videoconferencing.

#### Stable power

One of the most significant difficulties in establishing stable operation of the molecular diagnostics laboratory in Bo was inadequate power necessary to run laboratory equipment. Due to the reliance on hydroelectric power generation in Sierra Leone, the availability of municipal electricity (supplied by Bo/Kenema Power Service – BKPS) varies throughout the year and power is mostly unavailable during the dry season spanning from November to April. Even when available, the electricity is of poor quality due to inadequate design of electrical grid and is not suitable for powering sensitive scientific equipment [26]. To overcome this problem an innovative hybrid power system composed two self-contained local grids with two hybrid power subsystems (230 V, 50 Hz and 120 V, 60 Hz) was designed and deployed [25,26]. The system combined solar power generation with battery storage and diesel generator backup to provide reliable power for both basic laboratory infrastructure (e.g. lights, air conditioners) as well as sensitive scientific instruments (e.g. PCR cyclers, GenChip scanner).

#### Personnel training

Training is another key component of a successful laboratory set up. The laboratory personnel have undergone 10-week training in basic molecular biology diagnostic techniques including various PCR, and resequencing microarray technologies at Naval Research Laboratory (NRL) located in Washington, DC. A follow up training was conducted in Bo by NRL personnel. Personnel from MHRL also obtained scanner maintenance and calibration training from Affymetrix to ensure proper functioning of the scanner since this was the most delicate instrument of the system and needed to be periodically recalibrated. The proficiency of trained personnel was successfully demonstrated by conducting the investigation of an outbreak at the poultry farm that is described below. In addition to initial training, the NRL personnel were remotely supporting MHRL scientists taking advantage of the Internet connected computer network and Skype based videoconferencing.

### Stabilization of field samples and molecular biology reagents

While the stable power and use of freezers and refrigerators with battery backup solved the problem of storing perishable reagents and samples in the laboratory, it did not address the issues related to preservation samples collected in the field. Transport of temperature sensitive reagents needed for diagnostic procedures was also a significant problem due to unavailability of commercial “dry-ice” refrigerated transport service in Sierra Leone. To overcome these difficulties we explored applicability of two technologies: FTA paper for field sample collection and transport as well as stabilization of molecular biology reagents by freeze drying or related techniques.

#### FTA paper

FTA paper is a well-established technology for ambient temperature preservation of nucleic acids and was designed to protect nucleic acids of the stored sample from degradation caused by nucleases, oxidation, UV light and other processes [35,36]. FTA paper also rapidly inactivates pathogens making the infectious samples safer to handle by untrained personnel [35-37]. While it was shown that FTA cards are able to adequately store DNA samples at room temperature for at least 17 years without significant degradation [38,39], only limited data is available on the stability of RNA on FTA paper, especially when stored at elevated temperatures [28,35,40]. A series of experiments was conducted to find out if FTA technology might be suitable for short-term preservation of samples containing RNA viruses at high ambient temperatures characteristic for a tropical country such as Sierra Leone. Influenza B was used as a model organism for testing. Three different concentrations of influenza B RNA were spotted on FTA Minicards and stored at various temperature conditions for 1–10 days. The higher concentration of RNA (10^6^ and 10^4^ copies/μl) was consistently detectable after 10 days even when incubated at the highest tested temperatures (Table 3 and Additional file 1: Figures S1-S3). Although the lower concentration of RNA (10^2^ copies/μl) was not detectable in more than half of the analyzed samples, the lack of detection did not seem to correlate with storage conditions. These results indicated that in addition to the established capability to maintain stable DNA, the FTA cards could also be used to collect and store RNA samples for a time frame sufficient to transport and test samples. Inconsistent results of recovery of low concentrations of influenza B from FTA paper most likely reflected the phenomenon of dilution of samples deposited on FTA or inefficient recovery rather than degradation of the RNA.

**Table 3:** **Efficiency of detection of Influenza B RNA stored on FTA paper incubated at elevated temperatures**^**1**^

**Influenza B RNA concentration (copies/microliter)**	**Duration of incubation (hours)**									**Incubation temperatures (°C)**
	**24**			**72**			**240**			
	**1**	**2**	**3**	**1**	**2**	**3**	**1**	**2**	**3**	
10^6^	+	+	+	+	+	+	+	+	+	20-25 (RT)
	+	+	+	+	+	+	+	+	+	30
	+	+	+	+	+	+	+	+	+	30-45 (outdoors)
10^4^	+	+	+	+	+	+	+	+	+	20-25 (RT)
	+	+	+	F	+	+	+	+	+	30
	+	+	+	+	+	+	+	+	+	30-45 (outdoors)
10^2^	−	−	−	+	+	F	−	F	F	20-25 (RT)
	−	−	−	F	F	−	−	−	−	30
	F	F	F	−	F	F	−	−	−	30-45 (outdoors)
10^6^ (control RNA)	+			+			+			−20 (freezer)
10^2^ (control RNA)	+			+			+			−20 (freezer)

^1^Each experiment for particular combination of sample concentration and temperature was run in triplicate. Plus sign indicates that RT-PCR detection produced strong band of expected size that was observed on a gel; F indicates a faint band and a minus sign indicates absence of an amplification product detectable by visual inspection on a gel.

#### Reagent stabilization

In an effort to overcome the problems with the delivery of temperature sensitive reagents to Sierra Leone, we made an attempt to design molecular diagnostic protocols taking advantage of ambient temperature stabilized reagents. Commercially available stabilized reagents were used for reverse transcription, PCR, and real-time PCR together with modified protocols optimized for use with these regents. However in case of RPM platform, the stabilized reagents were developed in-house since there were no commercially stabilized reagents available. These reagents were developed by adaptation of previously published methods [41-43]. Details of the protocols, commercial reagents tested and custom reagent composition and stabilization procedures are described in supplementary data (Additional file 2).

While these ambient-temperature-stabilized reagent sets were very stable in high ambient temperatures, the testing results showed that diagnostic assays using these reagents were significantly less sensitive than traditional reagents (data not shown). Due to financial and time constraints, optimization of the stabilized reagents was not pursued. While the stabilized reagents were not used for the subsequent epidemiological investigation, the reagent stabilization technology has a great potential to make the molecular diagnostics more accessible in developing countries by eliminating the cold chain, greatly lowering power requirements that are dominated by refrigeration equipment and making the diagnostic protocols significantly less complex and error prone [41-43] and should be further explored in future.

As an alternative, reagents were transported as carry-on “dry ice” package in accordance with all airline regulations. The packages were prepared with sufficient amount of dry ice for 48 hours and passed through x-ray examination. Testing conducted with these reagents showed no noticeable difference in performance. Since US based personnel overseeing Mercy Hospital travel regularly (every 1–2 months) to the site, it was possible to ship reagents to maintain operation of the molecular diagnostics laboratory for sustained periods of time.

### Poultry outbreak investigation

To test the operational capabilities of MHRL molecular diagnostics laboratory broad-range microbial detection assays were used to investigate an outbreak that occurred at one of the few commercial poultry farms located in Bo. The concern was that the outbreak might have been caused by a highly pathogenic influenza virus which might be potentially transmitted to farm workers or poultry kept by individual owners. This is a significant risk especially in developing countries where it is a common practice to keep chickens in close proximity to the household and potentially expose the whole families to the poultry pathogens.

Two different assays, RPM-Flu and RPM-TEI, were used to analyze the outbreak samples. The analysis strategy relied on using microarray based broad-range detection assay to analyze just a small percentage of collected samples and follow up using single specific PCR based assays for larger numbers of samples based on the results of microarray analysis. Therefore only 15 randomly selected samples (see methods for selection process details) out of a total of 40 pharyngeal swab samples collected in the farm located in Bo and preserved on FTA paper were tested using resequencing assays. For comparison, 15 additional samples were selected from 5 other farms located in the Freetown area which housed mostly healthy chickens were also analyzed using the same microarrays. The results of RPM microarray analysis of selected samples are summarized in Table 4 and described below.

**Table 4:** Results of pathogen detection using RPM-Flu 3.1 microarray

**Farm**	**Sample**	Most likely ID*
1	B1	*K. pneumoniae*, *P. stutzeri*
	B2	*K. pneumoniae*, *P. stutzeri*
	B3	*K. pneumoniae*
	B4	no detection
	B5	no detection
	B11	no detection
	B12	*K. pneumoniae*, *P. aeruginosa*, *Staph.* (*mecA* gene)
	B13	*K. pneumoniae*, *Pseudomonas*
	B14	*K. pneumoniae*, *P. aeruginosa*, *Staph.* (*mecA* gene)
	B15	*K. pneumoniae*, *P. putida*, *Staph.* (*mecA* gene)
	B25	*K. pneumoniae*, *Pseudomonas*
	B26	*K. pneumoniae*
	B27	*K. pneumoniae*, *P. putida*
	B28	*K. pneumoniae*, *P. aeruginosa*, *Staph.* (*mecA* gene)
	B35	no detection
2	H2	no detection
	H3	no detection
	H4	(*P. aeruginosa***or***M. catarrhalis*)
6	HA5	(*P. aeruginosa* or *M. catarrhalis*)
	HA6	no detection
	HA7	*Pseudomonas*
5	A1	no detection
	A2	*P. putida*
	A16	no detection
9	J2	*P. aeruginosa*, *E. sakazakii*
	J7	no detection
	J10	no detection
4	W3	*K. pneumoniae*
	W4	*P. aeruginosa*
	W6	(*Pseudomonas ***or***Moraxella ***or ***Methylobacillus*)

*Most likely ID was determined using CIBSI algorithm and based on similarity analysis of the sequences obtained from the microarray with sequences deposited GenBank at the time of conducting the analysis (April 2010). Abbreviations used: *K. pneumoniae* = *Klebsiella pneumoniae*, *P. stutzeri* = *Pseudomonas stutzeri*, *Staph*. = *Staphylococcus* spp., *P. aeruginosa* = *Pseudomonas aeruginosa*, *P. putida* = *Pseudomonas putida*, *M. catarrhalis* = *Moraxella catarhalis*, *E. sakazakii* = *Enterobacter sakazakii*. Organism names enclosed in parentheses denote result of hybridization of a single microarray tile, which cannot be unambiguously identified based on the obtained sequence.

#### Bacterial pathogens

While no viral pathogens were detected using RPM assays, a number of bacterial pathogens including *Klebsiella pneumoniae* and several *Pseudomonas* species were found in analyzed samples.

An assortment of closely related *Pseudomonas* species (*P. stutzeriP. aeruginosaP. putida*) or undefined *Pseudomonas* was detected in 16 of the 30 analyzed samples. The ubiquitous presence of *Pseudomonas spp.*, a known opportunist organisms colonizing the avian respiratory tract [44], indicated that it played no significant role in the outbreak. *K. pneumoniae* on the other hand was found mostly in the samples from farm in Bo (73%, 12 out of the 15 analyzed samples)*,* while only one sample from farms outside of Bo was positive for this organism (7%, Table 4). This result suggested a link between the outbreak and the presence of *K. pneumoniae*. Although *K. pneumoniae* is usually considered an environmental contaminant, it may sporadically cause embryo mortality, yolk sac infections and mortality in young chickens, turkeys and ostriches. In addition, the concurrent infection of young turkeys with *K. pneumoniae* is known to increase the severity of respiratory disease caused by other pathogens [44]. The presence of *K. pneumoniae* in the majority of outbreak samples suggested that it is an opportunist pathogen colonizing/coinfecting sick chickens and increasing the severity of infection caused by another (unidentified) pathogen. This notion was also supported by the fact that higher percentages of outbreak samples were testing positive for more than one pathogen simultaneously (60% vs. 6% of healthy samples).

In addition to previously mentioned bacteria, the RPM-Flu microarray also detected *mecA* (methicillin resistance) gene in four samples collected from the farm in Bo. The *mecA* gene is responsible for staphylococcal resistance to a broad range of β-lactam antibiotics [45]. While *mecA* carrying bacteria in chickens were reported before [46], it is unknown if the staphylococci carrying this gene were acquired by poultry from humans or the spread of this resistance mechanism was purely zoonotic. Nonetheless, the prevalence of *mecA* gene (at least 10% of analyzed samples collected in Bo) in the staphylococci colonizing/infecting the analyzed population of chickens is a cause of concern due to a potential of spread to humans and warrants further study.

#### Influenza virus

Lack of positive detection of influenza in all samples tested with RPM-Flu assay indicated that it is unlikely that the outbreak was caused by any known strain of influenza virus as it was initially suspected. To independently confirm the RPM-Flu assay and expand the results of influenza A detection, all 136 collected samples were analyzed using a published real-time RT-PCR assay [34]. This assay, amplifying a conserved segment of influenza matrix gene to detect influenza A regardless of serotype, also did not detect the presence of this virus in any of the analyzed samples (data not shown).

Based on the obtained results, it was impossible to exclude the possibility that the outbreak was caused by one of the major poultry respiratory pathogens (such as avian paramyxovirus type 2 [47] or avian metapneumovirus [48]) that are not represented on the RPM-Flu microarray, since this assay was targeted for detection of human pathogens.

Although, the conclusive determination of the cause of the outbreak and the roles of particular identified pathogens was not possible based on the available data, the outbreak investigation demonstrated potential usefulness of the broad-range microbial detection technology in future investigations. The recent emergence and spread of highly pathogenic avian influenza strains has raised concern about outbreaks in poultry farms. As a result, the local health authorities usually treat poultry farm outbreaks as a potential deadly threat to humans, and order all the birds in the affected farms to be culled as a precaution. This practice results in a very significant economic burden to farm owners in developing countries and it may be avoidable. In this study, the delay of completion of the molecular analysis (approx. 11 months) caused by problems associated with logistics of reagent delivery did not enable us to prevent the slaughtering of the flock, however, the study indicated that application of broad–spectrum microbial diagnostics might make it possible in future outbreaks. While cost of conducting a single RPM assay (approx. $100-200) is too high for routine diagnostics in resource-limited settings, the technology may be a cost effective way for national surveillance of avian influenza and other important human pathogens. The power of a single set of RPM assays allows detection of a panel of pathogens that would otherwise require a full national reference laboratory infrastructure, which would costs millions of USD. In the case of this project the total cost of setting up the laboratory to run RPM based assays was approximately $250 thousand, including the building for laboratory set up, power equipment, scientific instruments, telecommunication infrastructure and training.

## Conclusions

The project has shown that successful deployment and application of an advanced diagnostic technology in the conditions of low-resource tropical country is feasible. One of the most important outcomes of this effort was identification of a set of technologies that are needed to achieve this goal in an extremely challenging environment. Laying this groundwork will help us and others to build infectious pathogen diagnostic capacity in developing world in an efficient way by taking advantage of technological “leapfrogging”.

## Competing interests

APM, BL and DAS are inventors of four US patents, and one pending patent application that are related to RPM technologies. These authors also receive royalty payment from Tessarae LLC (Potomac Fall, VA, USA), which licenses the RPM technologies for commercial purposes.

## Authors’ contributions

RA and TAL were involved in study conception, data collection, and drafting of the manuscript. APM was involved in the study conception, data analysis, and critical manuscript revision. ASB and AJS were involved in GIS data collection and manuscript revision. DHJ and UB were involved in poultry sample collection, molecular analyses and manuscript revision. BRB was involved in FTA card testing, and drafting part of the manuscript. MA was involved in poultry sample collection, and manuscript revision. BMK was involved in study conception and manuscript revision. NCL, BL and TAL were involved in MHRL personnel training. BL was involved study conception, data analysis and critical manuscript revision. DAS was involved in study conception and manuscript revision. All authors have read and approved of the final manuscript.

## Supplementary Material

Additional file 1 Supplementary Data Figures. Click here for file

Additional file 2 **Supplementary Data [**[30,31,34,49]**]**. Click here for file
