# Supplementary material for: Leapfrog diagnostics: Demonstration of a broad spectrum pathogen identification platform in a resource-limited setting
Source: Health Res Policy Syst. 2012 Jul 4;10:22. doi: 10.1186/1478-4505-10-22 (PMC3418216; doi:10.1186/1478-4505-10-22)
Supplement: Additional file 1 — Supplementary Data Figures. [file 1478-4505-10-22-S1.docx]

Supplementary Data – Figures

**
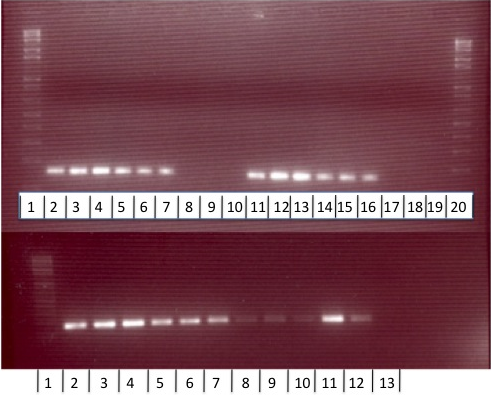
Figure S1: Comparison of FluB DNA samples following 24-hour incubation.** **Top gel**: Lanes 1 and 20 are a 1 kb DNA ladder; lanes 2-10 are FluB samples held at RT with lanes 2-4 = 10^6^ copies/µL, lanes 5-7 = 10^4^ copies/µL, and lanes 8-10 = 10^2^ copies/µL; lanes 11-19 are FluB samples held at 30 ºC with lanes 11-13 = 10^6^ copies/µL, lanes 14-16 = 10^4^ copies/µL, and 17-19 = 10^2^ copies/µL. **Bottom gel**: lane 1 is a 1 kb DNA ladder; lanes 2-10 are FluB samples stored outdoors at between 30-45 ºC, with lanes 2-4 = 10^6^ copies/µL, lanes 5-7 = 10^4^ copies/µL, and lanes 8-10 = 10^2^ copies/µL; lanes 11 and 12 are FluB samples stored at -20 ºC, containing 10^6^ and 10^2^ copies/µL, respectively; lane 13 = no template.


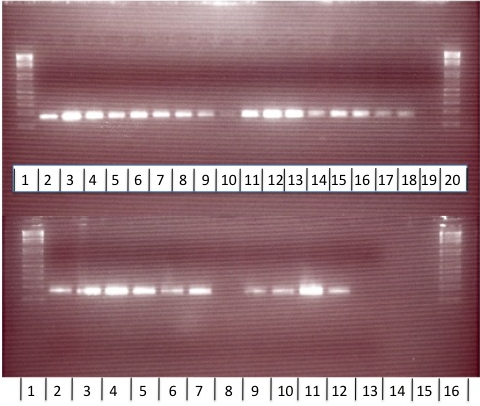
**Figure S2: Agarose gel containing FluB DNA samples following 3-day incubation.** **Top gel**: Lanes 1 and 20 are a 1 kb DNA ladder; lanes 2-10 are FluB samples held at RT with lanes 2-4 = 10^6^ copies/µL, lanes 5-7 = 10^4^ copies/µL, and lanes 8-10 = 10^2^ copies/µL; lanes 11-19 are FluB samples held at 30 ºC with lanes 11-13 = 10^6^ copies/µL, lanes 14-16 = 10^4^ copies/µL, and 17-19 = 10^2^ copies/µL. **Bottom gel**: lanes 1 and 16 are a 1 kb DNA ladder; lanes 2-10 are FluB samples stored outdoors at between 30-45 ºC, with lanes 2-4 = 10^6^ copies/µL, lanes 5-7 = 10^4^ copies/µL, and lanes 8-10 = 10^2^ copies/µL; lanes 11 and 12 are FluB samples stored at -20 ºC, containing 10^6^ and 10^2^ copies/µL, respectively; lane 13 = no template, lanes 14-15 – empty


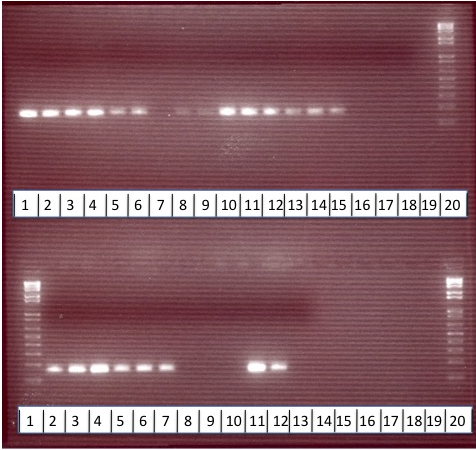


**Figure S3: Agarose gel containing FluB DNA samples following 10-day incubation.** **Top gel**: Lane 20 is a 1 kb DNA ladder; lanes 1-9 are FluB samples held at RT with lanes 1-3 = 10^6^ copies/µL, lanes 4-6 = 10^4^ copies/µL, and lanes 7-9 = 10^2^ copies/µL; lanes 10-18 are FluB samples stored outdoors at between 30-45 ºC, with lanes 10-12 = 10^6^ copies/µL, lanes 13-15 = 10^4^ copies/µL, and 16-18 = 10^2^ copies/µL. **Bottom gel**: lanes 1 and 20 are a 1 kb DNA ladder; lanes 2-10 are FluB samples held at 30 ºC, with lanes 2-4 = 10^6^ copies/µL, lanes 5-7 = 10^4^ copies/µL, and lanes 8-10 = 10^2^ copies/µL; lanes 11 and 12 are FluB samples stored at -20 ºC, containing 10^6^ and 10^4^ copies/µL, respectively; lane 13 = no template, lanes 14-19 empty.

**
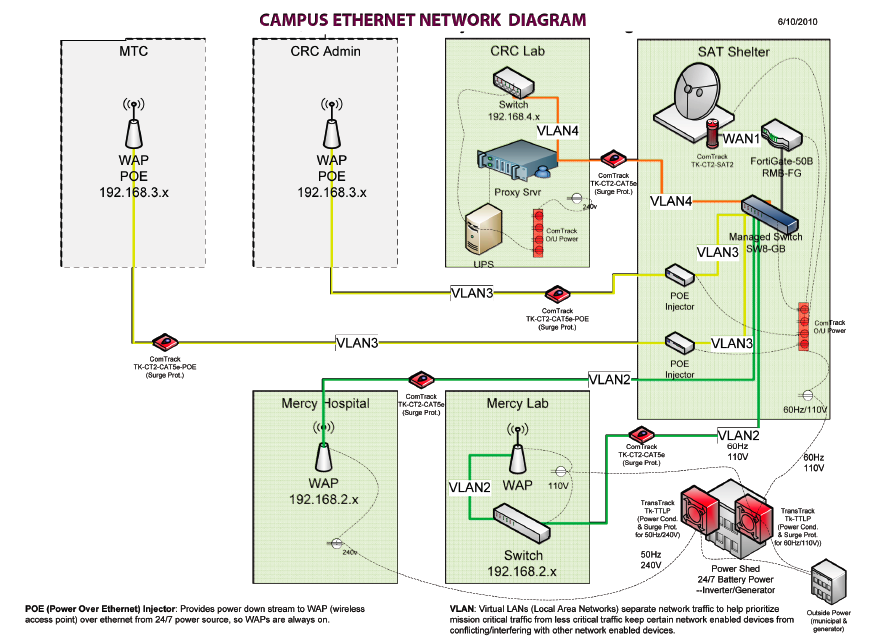
**

Figure S4
