# Supplementary material for: Leapfrog diagnostics: Demonstration of a broad spectrum pathogen identification platform in a resource-limited setting
Source: Health Res Policy Syst. 2012 Jul 4;10:22. doi: 10.1186/1478-4505-10-22 (PMC3418216; doi:10.1186/1478-4505-10-22)
Supplement: Additional file 2 — Supplementary Data [[30,31,34,49]]. [file 1478-4505-10-22-S2.docx]

**Supplementary Data**

**Development and testing of the ambient temperature stabilized reagents**

**Resequencing Pathogen Microarray (RPM) reagents.**

In order to develop an ambient temperature stabilized set of reagents for conducting resequencing pathogen microarray (RPM) protocols, a number of different stabilized reagents were tested by running the RPM detection protocol with a stabilized component used in place of a standard (ambient temperature labile) reagent. Commercially available stabilized reagents were tested as replacements in some stages of the RPM protocol, but for protocol steps that rely on custom primer mixes, in-house stabilized regent mixtures needed to be developed by lyophilization. For stages of RPM protocol where a commercial reagent was available, the performance of commercial reagent and in-house lyophilized mix were compared. Otherwise, only in-house prepared reagents were tested.

The compositions of the in-house prepared reagents are detailed in Table S1 (below). Most components including random primers, PCR primer mixes, and control templates are identical to those in the original RPM protocol [[1](#_ENREF_1)]. All departures from that protocol are explained below. Mixtures containing enzymes were supplemented with trehalose (Sigma, St. Louis, MO) in order to prevent the loss of enzymatic activity during the lyophilization process and stabilize enzymatic proteins in the dry form [[2](#_ENREF_2)]. One significant difference from the original protocol was the elimination of the final TdT based labeling step of the RPM sample-processing procedure. This was done due to the difficulty in successfully lyophilizing the TdT enzyme used for DNA fragment labeling (see Table S2). The removal of the TDT step required that the composition of the multiplex PCR mixture be modified from the original protocol to enable label incorporation at the multiplex PCR step. To achieve this, biotynylated dCTP was added to the mixture and the standard 50x nucleotide mix was replaced by a nucleotide cocktail containing 10mM of each of dATP, dGTP, dUTP and dCTP at the concentration of 1mM. All in-house prepared lyophilized reagents were prepared by mixing all of the components (on ice), deep-freezing the mixtures by placing in dry-ice bath for 10 minutes, and then transferring to a lyophilizer. The lyophilization was conducted at -70°C for 16 hours. Dry reagents were stored at ambient temperatures with desiccant.

Table S1.

Composition of the in-house developed, ambient temperature stabilized reagents

| **Name** | **Composition** |
| --- | --- |
| **NRL-mixRT1** (reverse transcription primer mix) | primer NLN (40μM) – 1μl  dNTP mix (10mM) – 1μl  NAC1 control (1pg/μl) – 1μl  TIM control (1pg/μl) – 1μl |
| **NRL-mixRT1+** (reverse transcription primer mix + control templates) | primer NLN (40μM) – 1μl  dNTP mix (10mM) – 1μl  NAC1 control (1pg/μl) – 1μl  TIM control (1pg/μl) – 1μl  Control templates^1^ (10^4^ copies/μl) – 2 μl |
| **NRL-mixRT2*** (reverse transcription enzyme mix) | 5x FS RT buffer – 4 μl  DDT (0.1mM) – 2 μl  RNase OUT – 1 μl (RNase inhibitor)  Superscript III – 1 μl (reverse transcriptase)  Trehalose (25%) – 1.5 μl |
| **NRL-mixPCR(1-4)** (multiplex PCR mixes – 4 versions) | 5x Go-Taq flexi buffer – 10μl  MgCl2 (25 mM) – 8 μl  Modified 50x dNTP mix^2^ – 2 μl  Biotin-14-dCTP (0.4 mM) – 5 μl  Primer NL (100 μM) – 1 μl  Primer multiplex^3^ 1, 2, 3 or 4 (1 μM) – 2.5 μl  GoTaq DNA polymerase (5U/μl) – 2 μl  Uracil-DNA Glycosylase (heat labile) (5U/μl) – 1 μl  Trehalose (25%) – 10 μl |
| **NRL-mixF** (fragmentation mix) | Tris pH 7.8 (10mM) – 6.6 μl  NEBuffer 4 – 3.3 μl  Affymetrix fragmentation reagent (DNaseI) – 0.1 μl  Trehalose (25%) – 2 μl |
| **NRL-mixHYB** | Hybridization Control DNA – 5μl  5 M TMAC – 72 μl  1 M Tris, pH 7.8 – 1.2 μl  1% Tween-20 – 1.2 μl  Herring Sperm DNA (10 mg/ml) – 1.2 μl  Acetylated BSA (50 mg/ml) – 1.2 μl  Control Oligo B2 – 2.8 μl |
| **NRL-mixL*** (labeling mix) | Biotin-N6-ddATP (conc) – 0.9 μl  TdT enzyme – 0.6 μl  Trehalose (25%) – 0.2 μl |

^1^Control template mixture included three different plasmid embedded synthetic DNA targets equivalent to Lassa virus GP, LP and NP protein coding genes at the concentration of 10^4^ targets/μl each. These synthetic constructs (LVVGP1, LVVLP1 and LVVNP) were previously used for validation of RPM-TEI v. 1.0 microarray and are described in detail in ref. [[3](#_ENREF_3)], Table S3.

^2^This nucleotide mixture contained dATP, dGTP, and dUTP at the final concentrations of 10mM and dCTP at final concentration of 1mM.

^3^Four separate multiplex PCR mixtures were prepared each containing a different primer multiplex. The compositions of these primer multiplex mixtures can be found in ref. [[3](#_ENREF_3)].

^*^ These lyophilized regents were prepared but not included in the final protocol for the following reasons: NRL-mixT2 was found to perform much worse than commercially available reagent for reverse transcription, NRL-mixL was found to be inactive as a result of TdT enzyme inactivation during the lyophilization and was eliminated by modifying the protocol to achieve labeling at the multiplex PCR step.

**Modified RPM protocol for use with stabilized reagents.**

Sample processing for RPM microarray hybridization were the same as previously described when using regular reagents [[1](#_ENREF_1)] with the following modifications.

1. During reverse transcription step NRL-mixR or NRL-mixR+ lyophilized reagent (containing primer and control template mixtures) and illustra Ready-To-Go RT-PCR Beads (containing reverse transcriptase enzyme) were used. To analyze an unknown sample, the analyzed sample and nuclease-free water were added to NRL-mixR to the final volume of 12μl and lyophilized regent was resuspended by gently pipetting up and down. To perform a control run NRL-mixR+, which contains additional control DNA template mix, was used in place of NRL-mixR and resuspended in 12μl of nuclease-free water. The resulting mixture was incubated at 65°C for 5 min. and then cooled on ice for at least 1 min. Next, one of the illustra Ready-To-Go RT-PCR Beads was resuspended in 20μl of nuclease-free water. Then, 8μl of the rehydrated reagent was added to the template and primer mixture prepared in the previous steps. The reverse transcription was conducted by incubating the reaction mixture at the following conditions: 10 min. at 25°C, 50 min. at 42°C and 5 min at 95°C.
2. The multiplex PCR step for each sample uses four lyophilized mixes: NRL-mixPCR(1-4) (containing PCR reagents with four different primer cocktails) were each resuspended in 45μl of nuclease-free water. Five microliters of reverse transcription mixture was added to each of the multiplex PCR mixes (to obtain total volumes of 50μl). PCR amplification and amplified product purification was conducted exactly as in the original protocol.
3. During the fragmentation step, lyophilized NRL-mixF was rehydrated by adding 10μl of nuclease-free water. Purified amplification products (25μl) were added to the fragmentation mix and processed as specified in the original protocol.
4. The separate labeling step was eliminated from the protocol. The labeling was achieved during the PCR amplification step.
5. The lyophilized hybridization solution (NRL-mixHYB) was rehydrated by adding 96μl of nuclease-free water. To prepare the sample for hybridization the fragmented sample was mixed with hybridization solution and processed further as in the original protocol.

**Testing results for the stabilized RPM reagents.**

Table S2 (below) shows the detailed results of RPM reagent testing. There are several steps in the sample processing before samples are hybridized to RPM and additional steps performed to label and detect hybridizing fragments. Testing involved substituting a stabilized reagent for a regular reagent at particular steps of the RPM protocol and assessing the performance by resequencing results of the control templates. The stages of RPM protocol for which the testing was conducted are specified in column “reaction”. Some reagents were tested multiple times to find out their performance after storage in a range of different temperatures (“storage” column) or/and to explore their performance with different types of templates (“test template” column).

**Table S2.**

Testing of ambient temperature stabilized reagents for conducting RPM protocol.

| Reaction^1^ | Reagent^2^ | Storage^3^ | Test template^4^ | Result^5^ |
| --- | --- | --- | --- | --- |
| RT | Bioneer-CRT | −20 | TIM | POS |
|  |  | 20-25 (RT) |  | POS |
|  |  | 35 |  | POS |
|  |  | 49 |  | POS |
|  | Bioneer-RT | −20 |  | NEG |
|  |  | 20-25 (RT) |  | NEG |
|  |  | 35 |  | NEG |
|  |  | 49 |  | NEG |
|  | GE-RTPCR* | 20-25 (RT) |  | POS |
|  |  |  | FluB | POS |
|  | NRL-mixRT2 |  | TIM | POS |
|  |  |  | FluB | NEG |
| PCR | Bioneer-PCR | −20 | TIM | POS |
|  |  | 20-25 (RT) |  | POS |
|  |  | 35 |  | NEG |
|  |  | 49 |  | NEG |
|  | NRL-mixPCR* | 20-25 (RT) |  | POS |
|  |  | 35 |  | POS |
|  |  | 50 |  | POS |
|  |  | 20-25 (RT) | Lassa4 | POS |
|  |  |  | Lassa2 | POS |
| FR | NRL-mixF* |  | TIM | POS |
|  |  |  | Lassa4 | POS |
|  | L-DNaseI |  | TIM | POS |
|  |  |  | Lassa4 | POS |
| LAB | NRL-mixL |  | TIM | NEG |
|  |  |  |  | NEG |
|  |  |  |  | NEG |
|  | NRL-mixPCR* |  |  | POS |
|  |  |  | Lassa4 | POS |
| HYB | NRL-mixHYB* |  | TIM | POS |
|  |  |  | Lassa4 | POS |
| ST | L-SAPE* |  | TIM | POS |
|  |  |  | Lassa4 | POS |
|  |  |  | Lassa2 | POS |

^1^Reaction of the RPM protocol: RT – reverse transcription, PCR – multiplex PCR, FR – fragmentation, LAB – labeling, ST – staining.

^2^Ambient temperature stabilized reagent used in place of standard reagents. See Table S3 for descriptions of reagents and Table S4 for the list of reagents selected for use in diagnostic protocols.

^3^Temperature in which the reagent was incubated prior to use for at least 4 days in the presence of desiccant.

^4^Template used to assess the microarray performance: TIM – standard RNA control template used in RPM protocol (see ref 2 for details), Lassa4 and Lassa2 – mixtures of 3 synthetic DNA templates containing sequences of Lassa virus genes at concentrations of 10^4^ copies/μl (Lassa4) and 10^2^ copies/μl (Lassa2), see Table S1, footnote 1 for detailed description of this synthetic Lassa template mix, FluB - clinical sample containing Influenza B virus of undetermined concentration but detectable by RPM-Flu microarray.

^5^Reagent testing results: POS – target sequence was detected and the quality of the sequence allowed for unambiguous identification, NEG – the target was not detected or the quality of the sequence did not allow correct identification.

* Reagents selected for final stabilized set of RPM regents.

**Other diagnostic protocols using stabilized reagents**

**One step RT-PCR for influenza A detection.**

The one step reverse transcription and PCR protocol (RT-PCR) was conducted using illustra Ready-To-Go RT-PCR Beads (GE Healthcare) and previously described PCR primers: MatrixF1 (5’- AAGACAAGACCAATYCTGTCACCTCT-3’) and MatrixR1 (5’- TCTACGYTGCAGTCCYCGCT-3’) [[4](#_ENREF_4)]. The final concentration of primers in the reaction mixture was 200 nM. The RT-PCR was performed according to manufacturers protocol with some modifications. In order to save reagents, the final reaction volume was reduced to 25μl as compared with 50μl in the original protocol. As a result, one bead of Ready-To-Go RT-PCR reagents was used to prepare reaction mix for two reactions. After adding the templates, the reaction mixture was incubated at the following conditions: 10 min at 25°C, 30 min at 42°C, 15 minutes at 95°C, 40 cycles of: 30 sec. at 96°C, 30 sec at 52°C and 60 sec. at 72°C. The amplification products were visualized on 1.2 agarose gels using FlashGel electrophoresis system and FlashGel camera (Lonza Rockland Inc., Rockland, ME)

**Two step real-time RT-PCR for influenza A detection**

The two step reverse transcription and real time PCR protocol (real-time RT-PCR) was conducted using AccuPower CycleScript RT PreMix (Bioneer), PerfeCta SYBR Green SuperMix (Quanta Biosciences) with the same primers as for RT-PCR protocol.

**Reverse transcription.** Primers, nuclease-free water and template (in the case of using liquid template preparation) were added to CycleScript RT PreMix to achieve 20 μl of total reaction volume. The final concentration of primers was 500nM. After resuspending the reagents, the FTA disk was added (in case of using FTA embedded template) and the mixture was incubated at the following conditions: 12 cycles of (1 min. 25°C, 4 min. of 50°C) and 5 min. of 95°C.

**Real-time PCR step.** 12.5 μl of PerfeCta SYBR Green SuperMix was combined with 2.5 μl of the RT product, primers (to concentration of 200nM) and nuclease-free water to obtain a final reaction volume of 25 μl. The reaction mixture was incubated at the following conditions: 40 cycles of: 15 sec at 95°C, 30 sec. at 52°C, 30 sec. at 72°C. The fluorescence data acquisition for SYBR 490 was conducted at the end of each cycle’s 72°C incubation.

**Table S3.**

Ambient temperature stabilized reagents tested

| **Abbreviation** | **Full name/description** | **Source** |
| --- | --- | --- |
| Bioneer-CRT | AccuPower CycleScript RT PreMix | Bioneer Corp., Alameda, CA |
| Bioneer-RT | AccuPower RT PreMix | Bioneer Corp., Alameda, CA |
| Bioneer-PCR | AccuPower PCR PreMix | Bioneer Corp., Alameda, CA |
| GE-RTPCR | illustra Ready-To-Go RT-PCR Beads | GE Healthcare Bio-Sciences Corp., Piscataway, NJ |
| QB-qPCR | PerfeCTa SYBR Green SuperMix | Quanta Biosciences, Inc., Gaithersburg, MD |
| NRL-mixRT1 | RT primer mix | in-house^1^ |
| NRL-mixRT1+ | RT primer mix + Lassa control templates | in-house |
| NRL-mixRT2 | reverse transcription enzyme mix | in-house |
| NRL-mixPCR(1-4) | multiplex PCR and biotin labeling mix | in-house |
| NRL-mixF | fragmentation mix | in-house |
| NRL-mixL | labeling mix | in-house |
| NRL-mixHYB | hybridization solution | in-house |
| L-SAPE | Streptavidin:R-Phycoerythrin | Columbia Biosciences Corp., Columbia, MD |
| L-DNaseI | DNase I, recombinant | Roche Diagnostics, Manheim, Germany |

^1^The compositions of reagents developed in-house is listed in Table S1.

**Table S4.**

Ambient temperature stabilized reagents selected for diagnostic protocols.

| **Protocol** | **Reaction** | **Reagent^1^** |
| --- | --- | --- |
| conventional RT-PCR | RT and PCR | GE-RTPCR |
| real-time RT-PCR | RT | Bioneer-CRT |
|  | real-time PCR | QB-qPCR |
| RPM^2^ | RT | NRL-mixRT1 or NRL-mixRT1+ |
|  |  | GE-RTPCR |
|  | multiplex PCR | NRL-mixPCR(1-4) |
|  | fragmentation | NRL-mixF |
|  | hybridization | NRL-mixHYB |
|  | staining | L-SAPE |

^1^See text and Table S1 above for compositions and method of stabilizing of in-house prepared reagents for RPM protocol.

^2^Only the reagents that differ from the original RPM protocol are shown. See the modified RPM protocol above for details.

References.

1. Lin, B., et al., *Using a resequencing microarray as a multiple respiratory pathogen detection assay.* J Clin Microbiol, 2007. **45**(2): p. 443-52.

2. Colaco, C., et al., *Extraordinary stability of enzymes dried in trehalose: simplified molecular biology.* Biotechnology (N Y), 1992. **10**(9): p. 1007-11.

3. Leski, T.A., et al., *Testing and validation of high density resequencing microarray for broad range biothreat agents detection.* PLoS ONE, 2009. **4**(8): p. e6569.

4. Carr, M.J., et al., *Development of a real-time RT-PCR for the detection of swine-lineage influenza A (H1N1) virus infections.* J Clin Virol, 2009. **45**(3): p. 196-9.
